# 786. Facility Reported vs. CLSI MIC Breakpoint Comparison of Carbapenem Non-susceptible (Carb-NS) *Pseudomonas aeruginosa* (PSA) From 2016-2019: A Multicenter Evaluation

**DOI:** 10.1093/ofid/ofab466.983

**Published:** 2021-12-04

**Authors:** Vikas Gupta, Kalvin Yu, Jason M Pogue, Janet Weeks, Cornelius J Clancy

**Affiliations:** 1 Becton, Dickinson and Company, Franklin Lakes, New Jersey; 2 College of Pharmacy, University of Michigan, Ann Arbor, Michigan; 3 University of Pittsburgh, Pittsburgh, PA

## Abstract

**Background:**

CLSI lowered *Pseudomonas aeruginosa* (PSA) Carbapenem (Carb) interpretive breakpoint minimum inhibitory concentrations (MICs) in 2012. It often takes several years for commercial test manufacturers and microbiology labs to incorporate revised breakpoints. We compare facility-reported rates of Carb-NS PSA to the 2012 CLSI MIC breakpoints, using a large nationwide database for isolates tested in 2016-2020 at United States (US) facilities.

Table. Imipenem (IPM)/meropenem (MEM)/doripenem (DOR) interpretation (evaluable isolates) results for PSA.

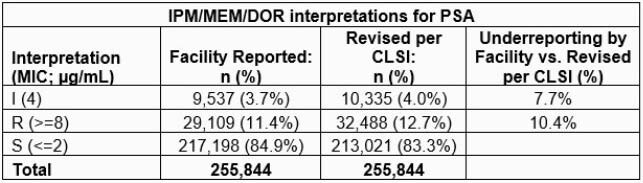

**Methods:**

All adults with a positive non-contaminant PSA culture (first isolate per 30-day period from blood, respiratory, urine, skin/wound, intra-abdominal, or other) in ambulatory and inpatient settings from 298 US hospitals from Q1 2016-Q4 2020 were evaluated (BD Insights Research Database, Becton, Dickinson & Company). Facility-reported Carb-non susceptible (NS) was defined as lab information system feed designations of susceptible (S), intermediate (I) or resistant (R) to imipenem (IPM), meropenem (MEM) and/or doripenem (DOR) per commercial panels. Where available, MICs were interpreted using CLSI 2012 Carb breakpoints (µg/ml) of ≤2 (S), 4 (I), ≥8 (R) for IPM/MEM/DOR. For evaluable PSA isolates we compared susceptibility results as reported by the facility to those using CLSI MIC breakpoints.

**Results:**

Overall, 86.9% (255,844/294,426) of non-duplicate PSA isolates with facility-reported IPM/MEM/DOR susceptibility interpretations also had interpretable MIC results. S rates were 84.9% and 83.3% as reported by facilities and determined by CLSI criteria, respectively (Table). Facilities under-reported Carb-NS by 9.8%, using CLSI criteria as the standard (10.4% and 7.7% of R and I isolates, respectively, were missed by facility reporting).

**Conclusion:**

Systematic application of CLSI breakpoints in 2016-20 would have had minimal impact on PSA S rates in the US. However, facility reporting failed to identify ~10% of Carb-NS isolates. The clinical implications of this observation are unknown. Facilities should know their local epidemiology, decide if under-reporting might be an issue, and assess if there is any impact on their patients.

**Disclosures:**

**Vikas Gupta, PharmD, BCPS**, **Becton, Dickinson and Company** (Employee, Shareholder) **Kalvin Yu, MD**, **BD** (Employee) **Jason M Pogue, PharmD, BCPS, BCIDP**, **Merck** (Consultant)**QPex** (Consultant)**Shionogi** (Consultant)**Utility Therapeutics** (Consultant)**VenatoRX** (Consultant) **Janet Weeks, PhD**, **Becton, Dickinson and Company** (Employee) **Cornelius J. Clancy, MD**, **Merck** (Grant/Research Support)

